# Role of dopamine in responsiveness to acute ethanol exposure in *Caenorhabditis elegans*

**DOI:** 10.17912/micropub.biology.001675

**Published:** 2025-07-02

**Authors:** Deepa Gayadin, McKenna Prunty, Stephanie JB Fretham

**Affiliations:** 1 Luther College, Decorah, Iowa, United States

## Abstract

Alcohol use and abuse is a common and prevalent disorder characterized by complex and individually variable physiological effects. *C. elegans* demonstrate multiple ethanol-induced behaviors and are an effective model for experimentally isolating environmental and genetic factors underlying the actions of ethanol. Using wild type and dopamine signaling mutant *C. elegans ,* the current study found that ethanol exposure results in dopamine release-dependent swimming induced paralysis and that dopamine influences acute sensitivity to ethanol. Taken together, the findings support a role for dopamine in mediating acute responses to ethanol.

**Figure 1. Role of dopamine in behavioral responses to acute ethanol exposure:**
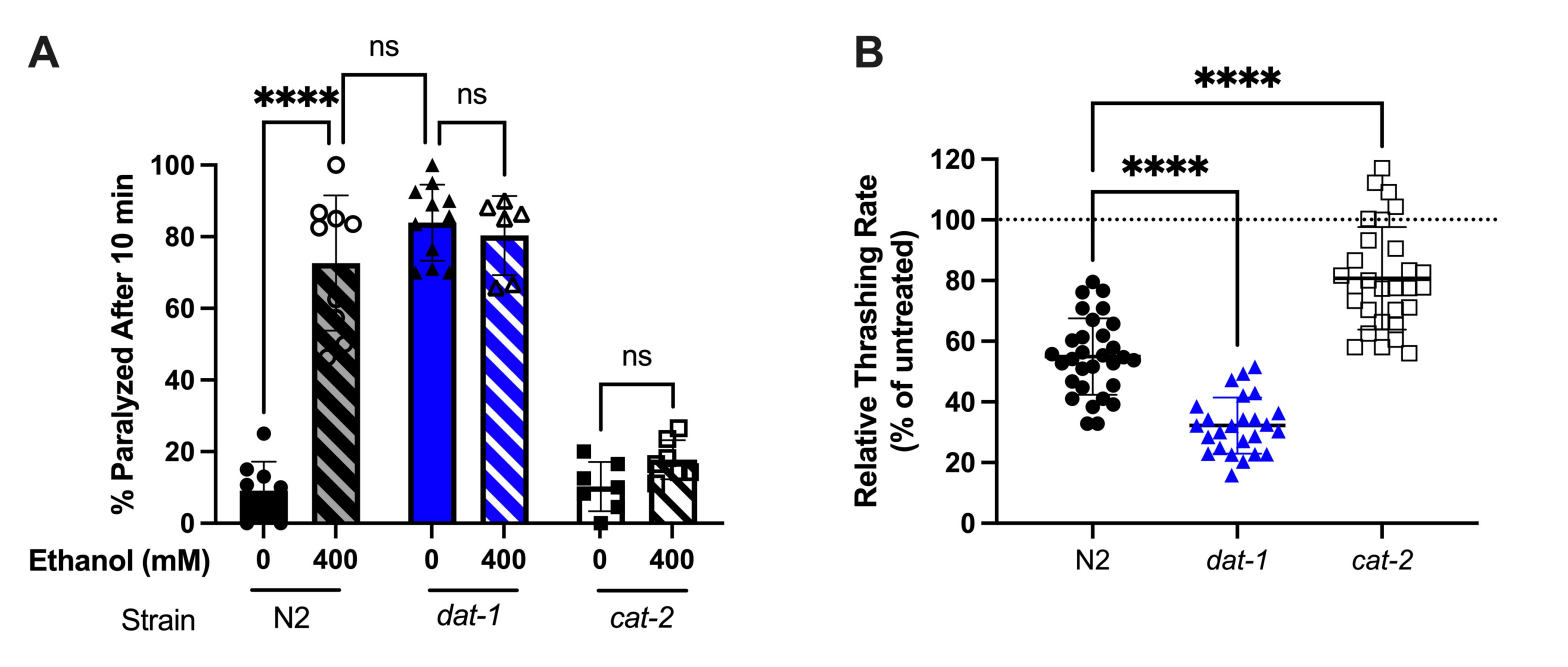
**A)** Acute exposure to 400mM EtOH in water for 10 minutes results in Swimming Induced Paralysis (SWIP) in wild type worms ( N2 ) but not in dopamine deficient worms ( *cat-2* ( e1112 )). Worms with increased extracellular dopamine ( *dat-1* ( ok157 )) have previously been shown to exhibit SWIP at similar levels (McDonald et al., 2007) and the paralysis rates are unchanged by EtOH. (n= 7-11 independent replicates consisting of 3-5 trials for each condition) **B)** 400mM EtOH in liquid NGM decreases thrashing rates relative to untreated controls (dashed line) in all worm strains after 10 minutes, however the reduction is enhanced in *dat-1* ( ok157 ) mutants and less severe in *cat-2* ( e1112 ) mutants relative to wild type N2 animals. (n=30 individuals/condition across 6 independent trials). Error bars indicate SEM, ANOVA and Tukey post-hoc were used for statistical comparisons: ****p<0.0001.

## Description

Alcohol (ethanol, EtOH) use and abuse is a common and prevalent disorder that affected approximately 28.9 million Americans in 2022 (National Survey on Drug Use and Health, 2023). Ethanol has complex physiological effects across multiple neurotransmitter systems that can vary greatly due to individual developmental, environmental, and genetic factors (Abrahao et al., 2017; Egervari et al., 2021). Dopamine is of particular interest due to its role in reward and addiction (Nutt et al., 2015). The nematode model organism *Caenorhabditis* *elegans* ( *C. elegans* ) exhibits multiple reversible and dose-dependent behavioral responses to ethanol exposure, in particular ethanol affects locomotion rates on solid and in liquid media (Davies, 2003; Johnson et al., 2013; Scholz, 2019). The *C. elegans* nervous system is extensively characterized including dopaminergic circuitry with high homology to mammalian systems of synthesis, vesicular packaging and release, receptors, and metabolism (Engleman et al., 2016; McMillen and Chew, 2024). Furthermore, *C. elegans* exhibit several easily observable dopamine-dependent phenotypes and provide an excellent system for examining the relationship between ethanol exposure and dopamine, though relatively few studies have addressed this relationship directly. Swimming and crawling locomotion patterns, particularly the transition from swimming to crawling, are altered by acute ethanol, an effect that may be mediated through dopamine signaling (Topper et al., 2014). In addition, dopamine may facilitate context-dependent learning involving extended exposure to ethanol cues (Bettinger and McIntire, 2004; Lee et al., 2009) and contribute to the development of functional tolerance during extended ethanol exposure in *C. elegans* (Pandey et al., 2021). However, the role of dopamine in acute responses to ethanol is unclear.

First, to determine if acute ethanol exposure alters extracellular dopamine, swimming induced paralysis (SWIP) was assessed in L4 animals exposed to 0 or 400mM EtOH in ddH _2_ O for 10 minutes ( **Fig. 1A** ). SWIP occurs when dopamine accumulates in the synapse due to excessive release or to ineffective clearance by the dopamine transporter dat-1 . Increased extracellular dopamine causes paralysis through prolonged activation of the dopamine receptor dop-3 on cholinergic motor neurons (McDonald, 2007). Notably SWIP is not observed in salty solutions such as NGM (Kudumala et al., 2019). In ddH _2_ O, 400mM EtOH significantly induced SWIP in wild type N2 animals compared to 0mM EtOH. The level of ethanol-induced SWIP in N2 animals was similar to observations in *dat-1 ( ok157 )* mutants. *cat-2* ( *e1112 )* dopamine-deficient tyrosine hydroxylase mutants had similar SWIP compared to N2 at 0mM EtOH, however 400mM EtOH did not induce SWIP. Taken together, these observations suggest that SWIP observed in N2 animals results primarily from ethanol-induced alterations in extracellular dopamine rather than a general paralytic effect of ethanol.

Second, to determine if dopamine contributes to the depressive locomotion effects of acute ethanol exposure, thrashing behavior was observed in N2 , *dat-1 ( ok157 )* , and *cat-2* ( *e1112 )* animals ( **Fig. 1B** ). In 0mM EtOH liquid NGM, which does not induce SWIP behavior, no paralysis was observed at 10 minutes and all strains maintained similar thrashing rates per 30s ( N2 =53.1±8.6; *dat-1 ( ok157 )* =51.9±6.0; *cat-2* ( *e1112 )* =51.5±6.8). Addition of 400mM EtOH reduced thrashing rates by 45% relative to untreated controls in N2 animals after 10 minutes, consistent with previous studies (Mitchell et al., 2007; Johnson et al., 2013). Ethanol reduced thrashing rates by 68% relative to untreated controls in *dat-1 ( ok157 )* mutants with increased extracellular dopamine, indicating increased sensitivity to acute ethanol. Conversely, thrashing rates were only reduced by 19% in dopamine deficient *cat-2* ( *e1112 )* animals, indicating reduced sensitivity to acute ethanol compared to N2 animals. Taken together, these observations suggest that dopamine has a central role in determining sensitivity to acute ethanol.

Collectively, these findings indicate that dopamine mediates acute behavioral change in response to ethanol exposure, potentially through ethanol-induced dopamine release or inhibition of dopamine clearance. The SWIP phenotype has been used to study other substances including amphetamine and cannabidiol (CBD) (Carvelli et al., 2010; Kudamala et al., 2019; Shrader et al., 2020), however to our knowledge, this study is the first to demonstrate SWIP in response to ethanol and provides an additional avenue for examining genetic and environmental variants that influence ethanol sensitivity.

## Methods

***C. elegans Maintenance***

All strains were obtained from the Caenorhabditis Genetics Center (University of Minnesota) and maintained at 20ºC on solid nematode growth medium (NGM) seeded with E. coli ( OP50-1 ). Age-synchronous animals from each strain were prepared by small-scale sodium hypochlorite treatment of gravid animals (Stiernagle, 2006) with resulting eggs maintained at 20ºC on seeded NGM plates for 3 days until reaching L4/young adult stage.

***Swimming Induced Paralysis (SWIP) Assay***

SWIP was measured as previously described (McDonald et al., 2007). Briefly, for each trial, 10 L4 animals were transferred from seeded NGM agar plates into 100μL of ddH _2_ O on a depression slide. Transfer time was less than one minute. Immediately following transfer, 5μL of 8.4M EtOH or ddH _2_ O was added to the slide to achieve a final EtOH concentration of 400mM. After 10 minutes, worms were scored for paralysis using a stereomicroscope. 7-11 independent replicates consisting of 3-5 trials each were measured for each strain and ethanol condition over multiple days by two different experimenters. SWIP was compared between ethanol conditions and across strains using 2-way ANOVA and Tukey's multiple comparisons in Prism (GraphPad).

***Thrashing Assay***

Thrashing was assessed according to Johnson et al., 2013. Five to seven L4/young adult worms were transferred into a depression slide containing 100µL liquid NGM and allowed to acclimate for 2-3 minutes. A baseline video recording was captured from a stereomicroscope before addition of 5µL of ddH _2_ O (control) or 8.4M EtOH to achieve 400mM EtOH. A second recording was taken 10 minutes following ethanol exposure. Thrashing, defined as head movement from one side to the other, was counted for 30 seconds to determine thrashing rates. 30 worms were assessed in each strain and ethanol condition across 6 individual replicates. Thrashing rates were compared using 1-way ANOVA and Tukey's multiple comparisons in Prism (GraphPad).

## Reagents

**Table:** 

**STRAIN**	**GENOTYPE**	**AVAILABLE FROM**
N2	*Caenorhabditis elegans* wild isolate	CGC
CB1112	*cat-2* ( *e1112 )* II	CGC
RM2702	*dat-1 ( ok157 )* III	CGC
